# Artificial intelligence to improve cytology performance in urothelial carcinoma diagnosis: results from validation phase of the French, multicenter, prospective VISIOCYT1 trial

**DOI:** 10.1007/s00345-023-04519-4

**Published:** 2023-07-22

**Authors:** Thierry Lebret, Xavier Paoletti, Geraldine Pignot, Mathieu Roumiguié, Marc Colombel, Laurent Savareux, Grégory Verhoest, Laurent Guy, Jérome Rigaud, Stéphane De Vergie, Grégoire Poinas, Stéphane Droupy, François Kleinclauss, Monique Courtade-Saïdi, Eric Piaton, Camelia Radulescu, Nathalie Rioux-Leclercq, Christine Kandel-Aznar, Karine Renaudin, Béatrix Cochand-Priollet, Yves Allory, Sébastien Nivet, Morgan Rouprêt

**Affiliations:** 1 Urology Department, Foch Hospital, Suresnes, France; 2 Institut Curie, Saint Cloud, France; 3 Université Versailles Saint-Quentin, Université Paris-Saclay, Saint Cloud, France; 4 Urology Department, Institut Paoli-Calmettes, Marseille, France; 5 Urology Department, Centre Hospitalier Universitaire (CHU) Rangueil, IUCT Oncopole, Toulouse, France; 6 Urology Department, Hôpital Edouard Herriot, Lyon, France; 7 Urology Auvergne Centre, Clinique de la Chataigneraie, Beaumont, France; 8 Urology Department, CHU Pontchaillou, Rennes, France; 9 Urology Department of Urology, CHU Gabriel Montpied, Clermont-Ferrand, France; 10 Urology Department, CHU Hôtel-Dieu, Nantes, France; 11 Urology Department, Clinique Beausoleil, Montpellier, France; 12 Urology Department, CHU Caremeau, Nîmes, France; 13 Urology Department, CHU Besançon, Besançon, France; 14 Pathology and Cytology Department, CHU Toulouse, IUCT Oncopole, Toulouse, France; 15 Centre de Pathologie Est, Hospices Civils de Lyon, Hôpital Femme-Mère-Enfant, Bron, France; 16 Service d’Anatomie et Cytologie Pathologiques, Hôpital Foch, Suresnes, France; 17 Department of Pathology, CHU Pontchaillou, Rennes, France; 18 Laboratoire d’Anatomie et Cytologie Pathologique, CHU Hôtel Dieu, Nantes, France; 19 Department of Pathology, CHU Hôtel Dieu, Nantes, France; 20 Centre de Recherche en Transplantation et en Immunologie, UMR 1064, INSERM, Université de Nantes, Nantes, France; 21 Department of Pathology, Hôpital Cochin, Assistance Publique-Hôpitaux de Paris, Paris, France; 22 Department of Pathology, Institut Curie, Saint-Cloud, France; 23 Institut Curie, PSL Research University, CNRS, UMR144, Equipe Labellisée Ligue Contre le Cancer, Paris, France; 24 VitaDX International, Rennes, France; 25 Urology Department, GRC n°5, Predictive ONCO-URO, Pitié-Salpêtrière Hospital, AP-HP, Sorbonne University, Paris, France

**Keywords:** Urothelial, Cancer, Bladder, Artificial intelligence, Deep learning, Markers

## Abstract

**Purpose:**

Cytology and cystoscopy, the current gold standard for diagnosing urothelial carcinomas, have limits: cytology has high interobserver variability with moderate or not optimal sensitivity (particularly for low-grade tumors); while cystoscopy is expensive, invasive, and operator dependent. The VISIOCYT1 study assessed the benefit of VisioCyt^®^ for diagnosing urothelial carcinoma.

**Methods:**

VISIOCYT1 was a French prospective clinical trial conducted in 14 centers. The trial enrolled adults undergoing endoscopy for suspected bladder cancer or to explore the lower urinary tract. Participants were allocated either Group 1: with bladder cancer, i.e., with positive cystoscopy or with negative cystoscopy but positive cytology, or Group 2: without bladder cancer. Before cystoscopy and histopathology, slides were prepared for cytology and the VisioCyt^®^ test from urine samples. The diagnostic performance of VisioCyt^®^ was assessed using sensitivity (primary objective, 70% lower-bound threshold) and specificity (75% lower-bound threshold). Sensitivity was also assessed by tumor grade and T-staging. VisioCyt^®^ and cytology performance were evaluated relative to the histopathological assessments.

**Results:**

Between October 2017 and December 2019, 391 participants (170 in Group 1 and 149 in Group 2) were enrolled. VisioCyt^®^’s sensitivity was 80.9% (95% CI 73.9–86.4%) and specificity was 61.8% (95% CI 53.4–69.5%). In high-grade tumors, the sensitivity was 93.7% (95% CI 86.0–97.3%) and in low-grade tumors 66.7% (95% CI 55.2–76.5%). Sensitivity by T-staging, compared to the overall sensitivity, was higher in high-grade tumors and lower in low-grade tumors.

**Conclusion:**

VisioCyt^®^ is a promising diagnostic tool for urothelial cancers with improved sensitivities for high-grade tumors and notably for low-grade tumors.

**Supplementary Information:**

The online version contains supplementary material available at 10.1007/s00345-023-04519-4.

## Introduction

Bladder cancer (BC) is a heterogeneous disease that includes de novo and recurrent low-grade subtypes with indolent disease evolution, and high-grade subtypes with recurrent, progressive, and potentially lethal outcome. Today, the gold standard for diagnosis and surveillance combines urinary cytology with white-light cystoscopy. If a tumor is suspected by cystoscopy, then final diagnosis and pathological staging are based on cystoscopy with biopsy [1]. However, cytology and cystoscopy are limited. Cytology, although noninvasive with high specificity, has high interobserver variability [2] with moderate sensitivity, particularly for low-grade tumors [3]. Cystoscopy is expensive, invasive, and operator dependent [1]. It detects papillary lesions with high sensitivity but has difficulties differentiating non-papillary and flat lesions in inflammatory lesions. Furthermore, cystoscopy can induce several potential side effects, including urinary tract infections, dysuria, hematuria, and perforation of the bladder wall [4].

These limits have driven the development of new noninvasive diagnosis tools [4–7]. In recent years, the use of artificial intelligence (AI) in medicine has expanded, providing opportunities for early detection and management of diseases [8]. AI is a powerful resource for building reliable models from complex and accumulating medical data using machine learning, algorithms, and artificial neuron networks [9]. AI has been successfully applied to urology for diagnosing, staging, treatment, and monitoring of some genitourinary malignancies [10]. AI has for example been used to interpret diagnostic imaging, histopathology, and genomic annotations.

To improve diagnosis and surveillance of patients with BC mainly non-muscle invasive bladder cancer (NMIBC), VitaDX (VitaDX International, France) developed a digital medical device (VisioCyt^®^) that uses deep learning-based automated image processing to analyze urothelial cell morphology [11]. The VisioCyt^®^ device detects bladder tumor cell aspects, in both high- and low-grade tumors cells, in smears from voided urine samples, by analyzing the morphological modifications of cell nuclei. Analysis of urine samples using VisioCyt^®^ is a practical, noninvasive alternative either alone or combined with cytology and cystoscopy.

The VISIOCYT1 trial investigated this innovative approach for detecting BC. The trial comprised an initial phase to develop and evaluate the algorithm and a second to validate the clinical performances of the VisioCyt^®^ diagnostic test. The results of the first stage have been reported [11]. We herein report the results of the second phase.

## Methods

The study design, slide preparation, and cell annotation for developing the algorithm, and the automated computed processing have been published [11]. The algorithm was developed from a database of thousands of urothelial cells selected and labelled, by pathologists and cytotechnicians, to identify and classify the various cell types, notably to distinguish normal cells from tumor cells. The global slide annotation used to train the algorithm was based on the results of the ‘gold-standard’ examinations. Patient with a negative cytology (negative for high-grade urothelial carcinoma or atypical urothelial cells) and a negative endoscopy were considered as having negative slides, and those with histologically confirmed urothelial neoplasia as having positive slides. Overall, VisioCyt^®^ is an in vitro diagnostic medical device that uses whole-slide digitalization and AI algorithms to identify tumor cells. The device performs a morphological analysis of voided urothelial cells, including the shape, size, and color of the nuclei. Below, we highlight important details pertaining to the clinical validation phase of the trial.

### Study design and patient selection

VISIOCYT1 was a French, multicenter, prospective, clinical trial conducted in 14 centers.

Patients older than 18 years of age with bladder endoscopy for suspected BC (either de novo or relapsed) or for exploring the lower urinary tract for other reasons were eligible. Patients with urinary tract infections, urinary lithiasis, prior pelvic radiotherapy or renal transplants were ineligible. Patients with positive cystoscopy or negative cystoscopy with positive cytology were included in Group 1: patients with BC. Those with negative cystoscopy and negative cytology were included in Group 2: patients without BC.

All study visits were performed according to standard of care. At Visit 1, eligible patients provided urine samples (50 mL) for VisioCyt^®^ diagnostic testing and performed cytology and cystoscopy. At Visit 2, histopathological analyses of tissues from cystoscopy with transurethral resection (TUR) or biopsies for patients in Group 1. Follow-up visits with cytology and cystoscopy were performed at 6 months (Visit 3) and 12 months (Visit 4). All assessments and additional visits were at the investigator’s discretion.

The study was approved by an independent Ethics Committee, “CPP Ile de France VIII—Hôpital Ambroise Paré”. All participants provided written informed consent before study participation. The study is registered at ClinicalTrials.gov (NCT02966691).

### Urine sample preparation

The natural void urine samples were collected at Visit 1, before cystoscopy. The urine samples were fixed with ThinPrep^®^ Cytolit, homogenized, and divided into two samples. One for routine cytology and one for the VisioCyt^®^ test (centralized at the Foch Hospital, Suresnes, France).

### Standard cytology

At the centers, the slides for cytology were evaluated by trained pathologists. All pathologists attended a 1-day training to obtain consensus on the use of the Paris System 2016 (TPS 2016) criteria [12] for urinary cytology. Urinary cytology was done using transmission microscopy, by standard routine procedures, before the histological results were disclosed. Patients with cytology slides with < 15 urothelial cells were considered as not evaluable and excluded from analyses.

### VisioCyt^®^ diagnostic testing

The slides prepared according to the VisioCyt^®^ protocol were scanned on three planes, digitized on brightfield with a dedicated scanner (Hamamatsu S60), and stored on an image sharing web server. Patients with slides containing less than 15 urothelial cells were excluded from the analysis. During the diagnostic validation phase of the VISIOCYT1 trial, digitized slides were analyzed and interpreted automatically by the VisioCyt^®^ device, without evaluations by pathologists and blinded to the histopathological results of the samples.

### Standard histopathological assessment

The histopathological analysis of bladder tissue, from cystoscopy with transurethral resection (TUR) or biopsy, performed at Visit 2, was based on the 2009 TNM classification of BC [13].

### Objectives and endpoints

The primary objective of the clinical validation phase of the study was to assess the diagnostic performance of the VisioCyt^®^ test, in terms of sensitivity. The performance of the VisioCyt^®^ test would be considered validated if the sensitivity was above 80% and that the lower bound of the 95% CI was above the 70% threshold. The secondary endpoints included the specificity of VisioCyt^®^ test, with specificity above 85% and that the lower bound of the 95% CI was above the 75% threshold. Furthermore, sensitivity of the VisioCyt^®^ test was also assessed in the following categories: grade of tumor differentiation (low- and high-grade tumors), T-staging within low- and high-grade tumors, and the trial groups. When appropriate, the performance of VisioCyt^®^ was compared with standard cytology. The sensitivity of standard cytology and of the VisioCyt^®^ diagnostic test were calculated relative to the histopathological assessments, performed at Visit 2. The specificity of standard cytology and the VisioCyt^®^ diagnostic test were calculated relative to data from patients in Group 2: those initially with negative cystoscopies and cytologies.

### Statistical analysis

Patient demographic and disease-related data were described using mean with standard deviation (SD), median with interquartile range (IQR), and/or numbers with percentages. Sensitivity was calculated from the number of patients positive to the diagnosis test (either cytology or VisioCyt^®^) among patients with BC (Group 1) and confirmed with an initial positive histology (at Visit 2). The specificity calculated from the number of patients negative to the diagnosis test among participants without BC (Group 2) confirmed with a negative cytology and cystoscopy (at Visit 2).

To respond to the primary objective, with 80% power for the superiority testing of sensitivity, relative to the theoretical threshold of 70%, and with expected sensitivity of 80%, 155 evaluable patients were required in Group 1. The primary endpoint analysis was planned in individuals allocated Groups 1 and 2. Similarly, to evaluate specificity with 80% power for the superiority testing, with a threshold of 75%, and with expected specificity of 85%, 145 evaluable individuals were required in Group 2.

The sensitivity and specificity were estimated with their confidence interval (CI) at 95% according to the Wilson method. The sensitivity and specificity of the VisioCyt^®^ test were compared using the Wald test with a one-sided alpha level of 2.5%. The performances of the VisioCyt^®^ and standard cytology tests were compared using the McNemar test.

The statistical analyses were performed using SAS (version 9.4 or more recent).

## Results

### Patient characteristics

**Fig. 1:**
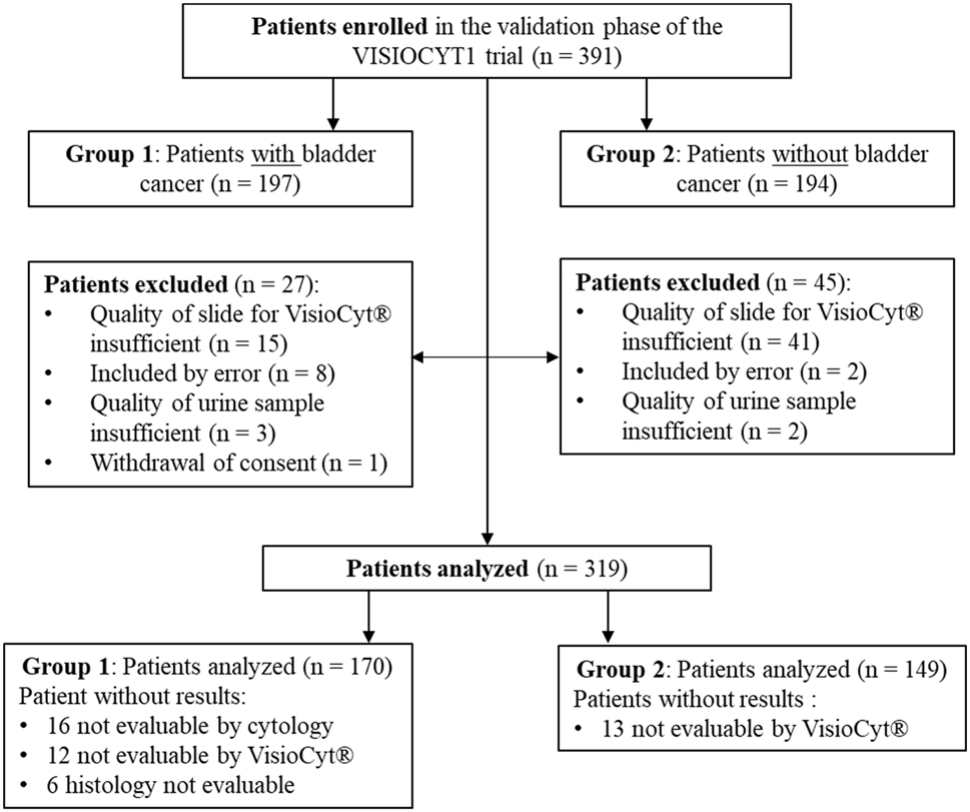
Patient flow diagram for the validation phase of the VISIOCYT1 trial

Between October 2017 and December 2019, 391 patients were enrolled in the clinical validation phase of the VISIOCYT1 trial. Among these, 319 were analyzed: 170 in patients with BC (Group 1) and 149 without BC (Group 2). The patient flow diagram for the trial is shown in Fig. 1.

Patients were mainly male, 132 (77.6%) in Group 1 and 91 (61.1%) in Group 2. The median age was 70 years (IQR 63–75) in Group 1 and 67 years (IQR 60–73) in Group 2. In Group 1, 137 patients (82.5%) had NMIBC (T-stages Ta, Tcis, and T1 [14]). Further baseline characteristics of patients in Group 1 are shown in the Supplementary Information section (Table S1).

### Diagnostic performance of the VisioCyt^®^ diagnostic test

The results of the analysis of the diagnostic performance of the VisioCyt^®^ test are summarized in Table 1. The overall sensitivity and specificity were analyzed in patients allocated Group 1 (with BC) and Group 2 (without BC). The overall sensitivity (primary outcome) of the VisioCyt^®^ test was 80.9% (95% CI 73.9–86.4%), and the lower bound of the 95% CI was significantly above the 70% threshold required for validation (*p* = 0.002). The VisioCyt^®^ test specificity was 61.8% (95% CI 53.4–69.5%), below the targeted lower-bound threshold of 75%. The sensitivity of the VisioCyt^®^ test was 93.7% (95% CI 86.0–97.3%) in high-grade tumors and 66.7% (95% CI 55.2–76.5%) in low-grade tumors. The sensitivity of the VisioCyt^®^ test in the subgroups (by tumor grade) according to T-staging were like those reported overall for high-grade tumors (96% for high-grade pTa and 95.8% for pT1) and low-grade tumors (66.2% for low-grade pTa).

**Table 1:** Analysis of the performance (sensitivity and specificity) of the VisioCyt^®^ test and standard cytology in the trial groups and in subgroups of Group 1, according to tumor grade and T-staging

Trial groups/subgroups analyzed	Histology at Visit 2	VisioCyt^®^ test results		VisioCyt^®^ test performance		Standard cytology results		Standard cytology performance	
		Negative, *n* (%)	Positive, *n* (%)	Sensitivity, % (95% CI)	Specificity, % (95% CI)	Negative, *n* (%)	Positive, *n* (%)	Sensitivity, % (95% CI)	Specificity, % (95% CI)
**Groups 1 and 2** (overall performance evalution)	Negative	84 (61.8)	52 (38.2)		61.8 (53.4–69.5)	149 (100)			100.0 (97.5–100.0)
	Positive	29 (19.1)	123 (80.9)	80.9 (73.9–86.4)		80 (54.1)	68 (45.9)	45.9 (38.1–54.0)	
**Group 1: Patients with bladder cancer (*n* = 170)**	Positive	29 (19.1)	123 (80.9)	80.9 (73.9–86.4)		80 (54.1)	68 (45.9)	45.9 (38.1–54.0)	
High-grade tumors	Positive	5 (6.3)	74 (93.7)	93.7 (86.0–97.3)		29 (37.2)	49 (62.8)	62.8 (51.7–72.7)	
T-staging									
Ta	Positive	1 (4.0)	24 (96.0)	96.0 (80.5–99.3)		12 (50.0)	12 (50.0)	50.0 (31.4–68.6)	
Tcis	Positive	1 (14.3)	6 (85.7)	85.7 (48.7–97.4)		4 (57.1)	3 (42.9)	42.9 (15.8–75.0)	
T1	Positive	1 (4.2)	23 (95.8)	95.8 (79.8–99.3)		6 (26.1)	17 (73.9)	73.9 (53.5–87.5)	
T2	Positive	1 (5.6)	17 (94.4)	94.4 (74.2–99.0)		4 (21.1)	15 (78.9)	78.9 (56.7–91.5)	
Low-grade tumors	Positive	24 (33.3)	48 (66.7)	66.7 (55.2–76.5)		51 (73.9)	18 (26.1)	26.1 (17.2–37.5)	
T-staging									
Ta	Positive	24 (33.8)	47 (66.2)	66.2 (54.6–76.1)		50 (73.5)	18 (26.5)	26.5 (17.4–38.0)	
**Group 2: Patients without bladder cancer (*n* = 149)**	Negative	84 (61.8)	52 (38.2)		61.8 (53.4–69.5)	149 (100)			100.0 (97.5–100.0)

*CI* confidence interval

### Diagnostic performance of standard cytology

The results of the analysis of the diagnostic performance of the standard cytology are summarized in Table 1. The overall sensitivity of standard cytology was 45.9% (95% CI 38.1–54.0%) and the specificity was 100.0% (95% CI 97.5–100.0%). The sensitivity of standard cytology was 62.8% (95% CI 51.7–72.7%) in high-grade tumors and 26.1% (95% CI 17.2–37.5%) in low-grade tumors. The specificity of standard cytology in Group 2 (patients without BC) was 100.0% (95% CI 97.5–100.0%).

### Diagnostic performance of the VisioCyt^®^ test compared to standard cytology

Overall sensitivity was significantly higher with the VisioCyt^®^ test, 80.9% (95% CI 73.9–86.4%), compared to 45.9% (95% CI 38.1–54.0%) with standard cytology, *p* < 0.0001 (McNemar test for paired comparison). Due to the trial’s design the specificity with the VisioCyt^®^ could not be compared to the specificity with the standard cytology. The trial was designed to only include patients with negative cytology in Group 2. Therefore, as expected, the specificity of standard cytology in Group 2 was 100.0% (95% CI 97.5–100.0%). Furthermore, the sensitivity of the VisioCyt^®^ test to detecting high-grade tumors was 93.7% compared to 62.8% with standard cytology, *p* < 0.0001. Similarly, the sensitivity of the VisioCyt^®^ test to detect low-grade tumors was 66.7% compared to 26.1% with standard cytology, *p* < 0.0001.

## Discussion

The overall sensitivity of the VisioCyt^®^ test was 80.9% (95% CI 73.9–86.4%) with the lower bound of the 95% CI, higher than the 70% threshold established for the trial to validate the VisioCyt^®^ test. In the subgroup analysis, the sensitivity of the VisioCyt^®^ test was 93.7% in high-grade tumors and 66.7% in low-grade tumors. However, the specificity was 61.8%, below the 75% threshold established.

Current standard surveillance comprises cytology and cystoscopy. The overall sensitivity of cytology was reported to be 48%: 84% for high-grade tumors and 16% for low-grade tumors with an overall specificity of 86% [3]. Cystoscopy has an overall sensitivity of about 56–70% and specificity of about 70–85% [15, 16]. In our trial, the VisioCyt^®^ diagnostic test had a sensitivity of 80.9% which was significantly higher than the 45.9% for standard cytology in our trial and the 48% reported [3]. In contrast, the 61.8% specificity of the VisioCyt^®^ test is lower than standard cytology, in our trial (100%) and reported (86%) [3]. In this trial, the sensitivity of standard cytology for high-grade tumors was 62.8%, lower than the 84% reported [3]. However, we used voided urine samples, with a lower concentration of cells, and not on bladder washings following cystoscopy. The specificity of the VisioCyt^®^ test compared to cytology, in our trial, was biased since all patients with positive standard cytology and those not evaluable were all allocated to Group 1, patients with BC. Thus, overall specificity of standard cytology in our trial was 100%, as expected.

VisioCyt^®^, compared to cytology from voided urine, has an improved sensitivity, for patients with low- and high-grade tumors. The role of VisioCyt^®^ in clinical practice may be useful for the surveillance of NIMBC and for BC diagnosis. Furthermore, VisioCyt^®^ should be useful for monitoring patients at high and very high risk of recurrence by reducing the number of cystoscopies. Fewer cystoscopies would potentially be more cost effective and reduce the risk of side effects. However, the utility of VisioCyt^®^ still needs to be confirmed in large prospective multicentric studies.

Thus, VisioCyt^®^ that uses voided urinary samples with analysis by a fully automated AI is a valuable addition to the diagnostic tools for detecting urothelial carcinoma. VisioCyt^®^ can be performed whenever a urine sample is available, it may be useful before cystoscopy and when lesions are not visible, and cystoscopy is not conclusive. In these cases, VisioCyt^®^ combined with cytology and complementary examinations, such as computed tomography and magnetic resonance imagery, may avoid repeating of time-consuming cystoscopy.

AI has been extensively applied to several urological conditions [17], including diagnosis of BC by cystoscopy [15, 18–20]. In contrast, few studies have investigated AI applications for diagnosing BC by cytology from voided urine [21–24]. To our knowledge, the VISIOCYT1 trial is the first prospective study to assess an AI-based diagnostic test for BC using urinary cytology. All, including our study, have highlighted the interest in using AI and image morphometry to improve the performance of diagnostic tools for BCs with increased sensitivity: overall and mostly in low-grade tumors [15, 18–20]. Low-grade tumor diagnosis is not reliable by cytology and the low-grade category has been excluded from the Paris System-2 classification [25, 26].

When evaluating diagnostic tools for BCs, it is critical that all suspicious and potentially malignant cells are identified [11, 18]. AI-based diagnostic tools, like VisioCyt^®^, have several advantages. First, they perform a complete morphological analysis of all urothelial cells present on the slide, thus identifying even the smallest of anomalies. Second, AI-based diagnostic tests ensure reproducibility and reliability of the results. Indeed, intra- and inter-observer variability observed with cytology can often be problematic. Finally, AI-based tools can learn, and it is expected that the performances of these tests will improve as more data are analyzed.

There are limitations to our work. The histological assessments were not centralized, thus there is potential interobserver variability. Furthermore, the standard cytology smears and the biopsy specimens were prepared according to each center’s standard practice. However, morphological cell appearance depends on the preparation technique. Patients included in Group 2 (patients without BC) of the VISIOCYT1 trial do not correspond to patients usually eligible for diagnostic testing.

To assess the benefit of using VisioCyt^®^ during the surveillance of patients with NMIBC at high and very high risk of recurrence, we are currently performing the DMIA study (NCT05176145).

## Conclusion

The VisioCyt^®^ test is a promising diagnostic tool for urothelial BCs. The test has improved sensitivity, compared to standard cytology, especially in low-grade tumors, but with lower specificity. VisioCyt^®^ is reproducible, reliable, and practical: requiring only easily accessible voided urine samples. VisioCyt^®^—performed before cystoscopy—may assist urologists during patient management, particularly during follow-up, to decide on the number and frequency of cystoscopy and other examinations.

## Supplementary Information

Below is the link to the electronic supplementary material.

Supplementary file1 (DOCX 15 KB)

## Data Availability

The data supporting the study results will be provided, upon reason reasonable request, from the corresponding author.
