# Supplementary material for: Artificial intelligence to improve cytology performance in urothelial carcinoma diagnosis: results from validation phase of the French, multicenter, prospective VISIOCYT1 trial
Source: World J Urol. 2023 Jul 22;41(9):2381–8. doi: 10.1007/s00345-023-04519-4 (PMC10465399; doi:10.1007/s00345-023-04519-4)
Supplement: Supplementary file 1 — Supplementary file1 (DOCX 15 KB) [file 345_2023_4519_MOESM1_ESM.docx]

**Supplementary information**

**Table S1** Baseline characteristics of patients with bladder cancer (Group 1, n=170)

| **Baseline characteristics** | |  | **Group 1 (n=170)** |
| --- | --- | --- | --- |
| Age (years) | |  |  |
|  | Mean (standard deviation) |  | 68.8 (10.6) |
| Sex, n (%) | |  |  |
|  | Male |  | 132 (77.6) |
|  | Female |  | 38 (22.4) |
| History of smoking, n (%) | |  |  |
|  | Current smoker |  | 37 (21.6) |
|  | Never smoked |  | 35 (20.6) |
|  | Former smoker |  | 98 (57.6) |
| Previously treated with Bacillus Calmette-Guerin (n=47), n (%) | |  |  |
|  | Yes |  | 29 (61.7) |
|  | No |  | 18 (38.3) |
| Post-operative intravesical instillation (n=47), n (%) | |  |  |
|  | Yes |  | 16 (34.0) |
|  | No |  | 31 (66.0) |
| Tumor type (at visit 2), n (%) | |  |  |
|  | Non-evaluable |  | 6 (3.5) |
|  | No information |  | 3 (1.8) |
|  | pTx |  | 1 (0.6) |
|  | pTa |  | 105 (61.8) |
|  | pTa low grade | 79 (75.2) |  |
|  | pTa high grade | 26 (24.8) |  |
|  | Ptis |  | 8 (4.8) |
|  | pT1 |  | 24 (14.5) |
|  | pT2a |  | 17 (10.2) |
|  | pT2b |  | 4 (2.4) |
|  | pT3a |  | 1 (0.6) |
|  | pT3b |  | 1 (0.6) |
